# VIO-GO: optimizing event-based SLAM parameters for robust performance in high dynamic range scenarios

**DOI:** 10.3389/frobt.2025.1541017

**Published:** 2025-09-18

**Authors:** Saber Sakhrieh, Abhilasha Singh, Jinane Mounsef, Bilal Arain, Noel Maalouf

**Affiliations:** 1 Electrical Engineering and Computing Sciences Department, Rochester Institute of Technology, Dubai, United Arab Emirates; 2 Department of Computer Engineering, University of Sharjah, Sharjah, United Arab Emirates; 3 Electrical and Computer Engineering Department, Lebanese American University, Byblos, Lebanon

**Keywords:** visual inertial odometry, event SLAM, batch gradient descent, optimization, edge image, dynamic and low-light environments

## Abstract

This paper addresses a critical challenge in Industry 4.0 robotics by enhancing Visual Inertial Odometry (VIO) systems to operate effectively in dynamic and low-light industrial environments, which are common in sectors like warehousing, logistics, and manufacturing. Inspired by biological sensing mechanisms, we integrate bio-inspired event cameras to improve state estimation systems performance in both dynamic and low-light conditions, enabling reliable localization and mapping. The proposed state estimation framework integrates events, conventional video frames, and inertial data to achieve reliable and precise localization with specific emphasis on real-world challenges posed by high-speed and cluttered settings typical in Industry 4.0. Despite advancements in event-based sensing, there is a noteworthy gap in optimizing Event Simultaneous Localization and Mapping (SLAM) parameters for practical applications. To address this, we introduce a novel VIO-Gradient-based Optimization (VIO-GO) method that employs Batch Gradient Descent (BGD) for efficient parameter tuning. This automated approach determines optimal parameters for Event SLAM algorithms by using motion-compensated images to represent event data. Experimental validation on the Event Camera Dataset shows a remarkable 60% improvement in Mean Position Error (MPE) over fixed-parameter methods. Our results demonstrate that VIO-GO consistently identifies optimal parameters, enabling precise VIO performance in complex, dynamic scenarios essential for Industry 4.0 applications. Additionally, as parameter complexity scales, VIO-GO achieves a 24% reduction in MPE when using the most comprehensive parameter set (VIO-GO8) compared to a minimal set (VIO-GO2), highlighting the method’s scalability and robustness for adaptive robotic systems in challenging industrial environments.

## 1 Introduction

SLAM is a key technology in the autonomous navigation of robots, serving as a fundamental element for the operation of autonomous vehicles (Sahili et al., 2023). Over the past 2 decades, research in SLAM and Visual Odometry (VO), using cameras either independently or in conjunction with inertial sensors, has led to highly accurate and robust systems that continually improve in performance (Campos et al., 2021).

As the complexity of autonomous applications grows, new challenges require innovative VO and SLAM solutions that deliver precision and reliability in increasingly dynamic scenarios. Standard cameras, while effective in certain conditions, encounter difficulties in low-light environments or during rapid movement due to motion blur and constrained frame rates. Event cameras, asynchronous visual sensors that address these issues, have emerged as a promising alternative (Hadviger et al., 2023). Event-based SLAM offers distinct advantages by eliminating motion blur, supporting high dynamic range (HDR), and operating at higher frame rates. However, event cameras may struggle in scenarios with minimal relative motion, such as in stationary states, where standard cameras excel in offering instantaneous and comprehensive environmental data, particularly in low-speed, well-lit conditions (Vidal et al., 2018).

This complementary nature suggests a hybrid approach, combining event and standard cameras with an inertial measurement unit (IMU) to produce a reliable and precise VIO framework (Chen et al., 2023). By compensating for each sensor’s limitations, this integrated framework is versatile and adaptable to a broad range of environmental conditions and movement patterns. One such example is Ultimate SLAM (Vidal et al., 2018), which integrates traditional cameras, event frames, and IMU data to provide reliable state estimation even in challenging scenarios.

As event cameras transform the way visual data is captured, new methodologies are needed to handle and interpret this unique data effectively (Sahili et al., 2023). A major limitation involves adapting to the asynchronous and sparse output from event cameras, contrasting with the dense, synchronous images produced by conventional cameras. Consequently, traditional vision algorithms developed for frame-based image sequences cannot be directly applied to event data (Gallego et al., 2022).

To bridge this gap, several techniques have been presented to transform asynchronous event data into a synchronous format (Guan et al., 2023). Some methods directly process raw event streams without accumulating frames (Alzugaray and Chli, 2018). Others use learning-based methods to create intensity images from events (Gehrig et al., 2020). Another approach is the generation of motion-compensated event or edge images by grouping events within specific spatial and temporal windows, highlighting scene edges and providing a structured visual representation of event data (Vidal et al., 2018; Rebecq et al., 2017b). However, this method presents challenges, requiring substantial parameter adjustments specific to each environment, which becomes burdensome when transitioning across diverse scenarios (Huang et al., 2024). This dependency on manual tuning creates a bottleneck for VIO systems, which must exhibit robust performance in unfamiliar environments where the event count varies widely. For practical applications, especially within Industry 4.0, where environments are constantly changing, manual parameter adjustments become impractical (Mahlknecht et al., 2022).

**FIGURE 1:**
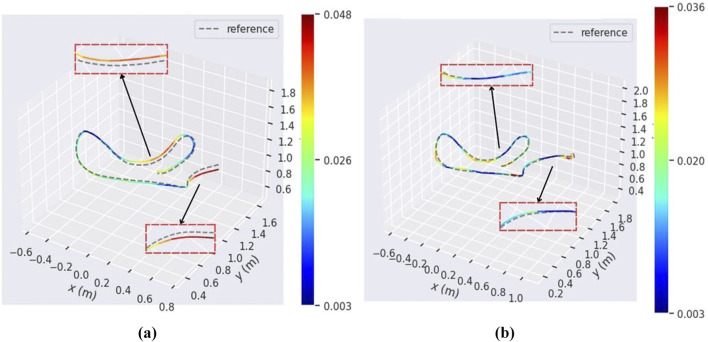
The estimated trajectory of Ultimate SLAM (Vidal et al., 2018) and VIO-GO aligned with the ground truth trajectory for the Hdr_boxes sequence in (Mueggler et al., 2017b). The figures show that VIO-GO produces a precise trajectory with a low absolute position error (APE) value, which is comparable to the trajectory estimated by Ultimate SLAM. **(a)** Estimated trajectory (fine-tuned parameters). **(b)** Estimated trajectory (VIO-GO).

In this paper, we present VIO-GO, a novel approach designed to enhance the performance and robustness of Event SLAM in unknown environments through targeted parameter optimization. Our method focuses on tuning parameters for Visual SLAM systems that use motion-compensated images to represent event data. By integrating event-based SLAM methods with a BGD algorithm, VIO-GO enables iterative refinement of parameters across diverse scenes with varying event generation rates. Unlike conventional motion-compensated image methods, VIO-GO minimizes the need for extensive manual parameter tuning, leading to improved time efficiency. Experimental results indicate that VIO-GO outperforms both fixed-parameter motion-compensated approaches and state-of-the-art EVIO methods, demonstrating superior performance across various dynamic scenes. Figure 1 shows a comparison between VIO-GO and Ultimate SLAM using its fine-tuned parameters, highlighting the improvements in performance achieved by VIO-GO.

## 2 Related work

The integration of RGB cameras and inertial sensors has long been foundational in VIO systems. However, recent advancements have seen an increasing shift towards the inclusion of event cameras, marking a pivotal development in the field of Visual SLAM. This literature review is divided into two subsections: Event-Based Visual-Inertial Odometry (EVIO) and Adaptive Parameter Optimization, providing a focused examination of the latest research.

### 2.1 Event-based visual-Inertial odometry (EVIO)

EVIO integrates the high-speed, high-contrast sensitivity of event cameras combined with inertial data from accelerometers and gyroscopes (Chen et al., 2023). Unlike traditional cameras, event cameras capture changes in a scene at up to 1 MHz, allowing them to handle rapid motion without issues like image blur, temporal aliasing, or saturation under intense lighting. These attributes make event cameras particularly useful for dynamic and low-light environments (Zhu et al., 2017).

The study in (Mueggler et al., 2017a) examines the integration of event frames and inertial data using a continuous-time model. However, real-time application remains challenging due to the computational burden of adjusting spline parameters for each incoming event. In a different approach, the authors in (Rebecq et al., 2017b) propose a real-time event-based VIO pipeline that uses optical flow estimation, grounded in the recent camera pose, scene configuration, and inertial data, to track visual features across multiple frames. These tracked features are subsequently combined with inertial measurements through keyframe-based nonlinear optimization. Similarly, in (Vidal et al., 2018), the authors integrate event streams, standard frames, and IMU data through nonlinear optimization process, achieving a notable accuracy enhancement of 130% over event-only frames and 85% over standard frames with IMU data. This system also supports real-time integration with a quadrotor. Furthermore, the authors in (Censi and Scaramuzza, 2014; Kueng et al., 2016) introduce low-latency, event-based VO techniques that accurately estimate rotation and translation using Dynamic Vision Sensors (DVS) alongside conventional CMOS cameras in natural scenes.

In (Kim et al., 2016), the authors discuss an event-based 6-degree-of-freedom (6-DoF) VO system, using three decoupled probabilistic filters to estimate the camera’s pose, a 3D scene model, and image intensity. However, this approach incurs a significant computational burden, requiring GPU use for real-time performance. In (Zhu et al., 2017), the EVIO method is presented, where an Extended Kalman Filter (EKF) fuses event data with pre-integrated IMU measurements, demonstrating the potential of event-based VIO for applications like planetary exploration. To further explore this potential (Mahlknecht et al., 2022), introduce the Event-based Lucas-Kanade Tracking VIO (EKLT-VIO), which integrates an event-based tracker developed by (Gehrig et al., 2020) in the front-end and a filter-based back-end to conduct VIO in Mars-like environments. Their results show a 32% improvement in MPE under low-light and HDR conditions compared to prior frame-based and event-based VIO techniques, although front-end and back-end parameter selection were not addressed. Expanding upon previous methodologies, PL-EVIO, proposed by (Guan et al., 2023), tightly integrates event-based point and line features, standard frame point features, and IMU data, thereby providing more geometric restrictions and enhancing robustness. While these advancements mark significant progress in Event SLAM, the literature reveals a gap in adaptive parameter optimization across various scenarios. Our approach aligns closely with methodologies in (Vidal et al., 2018; Rebecq et al., 2017b), which use motion-compensated images to represent event data but require significant manual parameter tuning for diverse environments. Our goal is to streamline the process by developing a pipeline capable of automating parameter optimization, thereby enhancing both the practicability and time efficiency of Event SLAM systems.

### 2.2 Adaptive parameter optimization

Integrating event cameras into VO/VIO systems presents a major challenge that arises from the asynchronous nature of event streams, which fundamentally differs from synchronous image data. Consequently, many methods designed for traditional image-based cameras cannot be directly applied to event-based systems. To address this gap, various techniques for representing event data have been introduced in the literature (Guan et al., 2023). Common approaches involve applying conventional feature detection and tracking methods to edge images created from motion-compensated event streams (Vidal et al., 2018; Rebecq et al., 2017b). However, these methods often require extensive parameter adjustments to adapt to specific fluctuations in event density, which can impact VIO system performance. Through a review of existing parameter optimization methods, we aim to identify the most effective strategies for enhancing event-based VIO systems and highlight areas where further optimization could improve system adaptability and reliability.

In (Li et al., 2020), the authors use a Stochastic Gradient Descent (SGD) approach for localization, coupled with scan matching via a 2D LiDAR system. This SGD-based approach enables the localizer to effectively track the robot’s state, generating a coherent trajectory of its movements. The technique attained a position error of 0.26 m and a heading error of around 5°. In (Torroba et al., 2023), the authors employ SGD to optimize the evidence lower bound (ELBO) on Gaussian process maps by estimating mini-batches, which allowed real-time performance on large-scale datasets and was successfully tested in a live Autonomous Underwater Vehicle (AUV) mission. Similarly, the authors in (Song et al., 2021) examine SGD for map classification in SLAM, while (Beomsoo et al., 2021) implements SGD to refine the policy network within the Proximal Policy Optimization algorithm. These approaches demonstrate the effectiveness of SGD in achieving both accuracy and efficiency for front-end and back-end optimization in dynamic environments.

In (Rebecq et al., 2017a), the authors use an intermediate representation by accumulating events into an edge-like image, employing a Gradient Descent (GD) approach that simplifies representation by randomly sampling pixels. This technique improves tracker speed and enhances robustness by increasing resilience to occlusions. In another approach, Luo et al. (2019) introduce a stage-wise SGD algorithm with a selective update mechanism to efficiently select a subset of training images for direct SLAM tracking, ensuring faster convergence.

As discussed, although significant research has focused on optimizing conventional SLAM methods, limited studies have applied GD approaches specifically to optimize Event SLAM parameters. Given the potential benefits, this work adopts the GD approach to optimize front-end and back-end parameters, particularly in challenging low-light and HDR scenarios.

## 3 Motion-compensated EVIO framework

This section details the motion-compensated event image state estimation framework, which serves as the backbone of the methodology presented in Section 4. Optimization of the state estimation parameters is addressed in the following paragraphs.

The motion-compensated EVIO system detects features within the edge image created from motion-compensated events by employing conventional image-based feature detection techniques. For example (Rebecq et al., 2017b), integrates event data with IMU data to obtain an accurate motion-compensated EVIO pipeline that leverages the distinctive features of event cameras to enable accurate state estimation in challenging scenarios. This approach is further extended in Ultimate SLAM (Vidal et al., 2018), where standard frames are incorporated as an additional sensing modality, achieving a more reliable and precise state estimation.

The motion-compensated EVIO system is traditionally divided into two parts: the front-end process, which processes a stream of events to establish feature tracks and triangulate landmarks, and the back-end, which integrates these feature tracks, landmarks, and IMU measurements to constantly update both current and past sensor states (Rebecq et al., 2017b). However, employing the edge image in this state estimation framework presents difficulties that often demand extensive parameter tuning.

To address these limitations, this work aims to enhance existing methods by developing an automated parameter optimization pipeline that facilitates the tuning process and identifies optimal parameters across diverse scenarios. The following paragraphs discuss key parameters that can be optimized within both the front-end and back-end components.

### 3.1 Front-end process

The main approach in the front-end is to generate event frames from spatiotemporal clusters of events, followed by applying feature detection and tracking techniques. This state estimation system builds on methodologies from (Vidal et al., 2018; Rebecq et al., 2017b), where features are detected and tracked within edge images derived from motion-compensated events, employing conventional image-based feature detection and tracking methods. Particularly, the FAST corner detector (Rosten and Drummond, 2006) and the Lucas-Kanade tracker (Lucas and Kanade, 1981) are utilized for these purposes. Additionally, features from standard frames are extracted and incorporated into the back-end optimization module, enhancing overall robustness and accuracy.

In noise-free scenarios, event frames can be represented as $e_{f}=\left(X_{f},t_{f},p_{f}\right)$ , where $X_{f}$ denotes the pixel value $\left(x_{f},y_{f}\right)$ , $t_{f}$ indicates the elapsed time, and $p_{f}$ signifies the polarity ranging from {-1,+1}. Additionally, the events $e_{f}$ are synchronized by aligning them with the spatio-temporal windows of events based on the timestamps of the conventional frames. For each conventional frame at time $t_{f}$ , a new spatiotemporal event window $W_{k}$ is defined as follows:

$$W_{k}=\left\{e_{j\left(t_{f}\right)-F_{S}+1};\ldots \ldots ;e_{j\left(t_{f}\right)}\right\}.$$ (1)

Here, $j\left(t_{f}\right)$ denotes the index of the first event with a timestamp $t_{j}<t_{f}$ , and $F_{S}$ represents the size of the window. Subsequently, each spatiotemporal event window undergoes a transformation into an artificial event frame $I_{k}$ by applying motion compensation at its individual timestamp, as demonstrated in the next equation:

$$I_{k}\left(x\right)=\underset{e_{r}\epsilon W_{K}}{\sum }\delta \left(x-x_{c}^{\prime }\right),$$ (2)

where δ (x) represents the Kronecker delta, $x_{c}$ denotes the adjusted event location acquired by shifting event $e_{r}$ to align with the specified event camera frame. Further, it is necessary to adjust the movement of every event locally based on its respective timestamp due to the limited information in small window sizes and the motion blur introduced by extensive window sizes. The $x_{c}^{\prime }$ in Equation 2 can be calculated using the formula given by (Rebecq et al., 2017b), as shown in Equation 3:

$$x_{c}^{\prime }=\pi_{o}\left(T_{tm,tn}\right)\left(Z\left(x_{c}\right)\pi_{o}^{-1}\left(x_{c}\right)\right),$$ (3)

where $x_{c}$ denotes the event pixel location, $\pi_{o}\left(:\right)$ is the event camera projection sample derived from previous inherent calibration, $\left(T_{tm,tn}\right)$ signifies the gradual transition of the camera poses at times $t_{m}$ and $t_{n}$ , derived from integrating the inertial measurements, and $Z\left(x_{c}\right)$ represents the scene depth at time $t_{f}$ estimated through a 2D linear interpolation.

The count of events $F_{S}$ in each spatiotemporal window needs to be adapted and can be optimized according to the texture density present in the scene. Hence, in this work, it has been chosen as one of the optimized parameters^1^. The median depth of the current landmarks $M_{D}$ can produce satisfactory results with reduced computational costs compared to linearly interpolating the depth $Z\left(x_{c}\right)$ . Therefore, the median depth of landmarks is optimized using the proposed method^1^ presented in Section 4.

New features are identified using the FAST corner detector, which is applied to both motion-compensated event frames and standard frames (Mueggler et al., 2017a). This approach ensures an even distribution of features across the image by using a bucketing grid:

$$S_{x\to y}=\left\{\begin{array}{cc} D,\; & \text{if }I_{x\to y}\leq I_{x}-T\\ S,\; & \text{if }I_{x}-T<I_{x\to y}<I_{x}+T\\ B,\; & \text{if }I_{x}+T\leq I_{x\to y} \end{array}\right.$$ (4)

where $I_{x}$ is the intensity at pixel x , T is the threshold value, $I_{x\to y}$ denotes the intensity difference between pixel x and y , D is the darker corner, S is the similar pixel, and B is the brighter corner. To effectively detect these features, the threshold of the FAST detector is optimized in this work^1^. These features are subsequently tracked from $I_{x}$ to $I_{x}+1$ , derived through an incremental transformation. Moreover, landmarks are tracked using the pyramidal Lukas-Kanade tracking algorithm (Lucas and Kanade, 1981), with the number of pyramid levels for feature extraction set as an automatically adjusted parameter^1^. Furthermore, a two-point RANSAC approach (Mueggler et al., 2014) is used for additional filtering of outlier feature tracks. In this system, the parameters for detection and tracking are maintained consistently across both motion-compensated event frames and conventional frames. Moreover, if the number of tracked features drops below a certain threshold $K_{T}$ , features are re-detected.

### 3.2 Back-end process

This section explores the integration of feature tracks from the event stream with IMU data, using a smoothing-based nonlinear optimization method on chosen keyframes. A detailed comprehensive analysis of IMU biases and kinematics can be found in (Rebecq et al., 2017b; Guan et al., 2023). The visual-inertial nonlinear optimization is described by a cost function $J_{\mathrm{V IO}}$ , which consists of three components: two weighted reprojection errors associated with event-based and conventional camera data, and an inertial error $E_{s}$ . The cost function $J_{\mathrm{V IO}}$ is formulated as shown in Equation 5:

$$J_{\mathrm{V IO}}=\overset{1}{\underset{i=0}{\sum }}\overset{K}{\underset{k=1}{\sum }}\underset{j\epsilon ȷ\left(i,k\right)}{\sum }E^{i,j,k^{T}}W_{r}^{i,j,k}E^{i,j,k}+\overset{K-1}{\underset{k=1}{\sum }}E_{s}^{k^{T}}W_{s}^{k}E_{s}^{k},$$ (5)

and the reprojection error $E^{i,j,k}$ is given by Equation 6:

$$E^{i,j,k}=z^{i,j,k}-\pi_{i}\left(T_{c_{is}}^{k}T_{sW}^{k}I^{i,j}\right).$$ (6)

In the previous equations, i represents the sensor identifier, k signifies the frame identifier, and j refers to the landmark. The set ȷ(i,k) includes the landmarks tracked by sensor i in the $k^{th}$ frame. The data matrix for each landmark measurement $I^{i,j}$ is represented as $W_{r}^{i,j,k}$ , while $W_{s}^{k}$ denotes the data matrix corresponding to the IMU error in the $k^{th}$ frame. Additionally, $z^{i,j,k}$ denotes the calculated image coordinates for every $k^{th}$ frame. The IMU error is computed as the disparity between predicted and actual trajectories (Leutenegger et al., 2013). Optimization is performed selectively, focusing on a subset comprising of keyframes and the last K frames in a sliding window, while predictions for intervening frames are propagated using IMU data. The number of keyframes employed in the back-end process is one of the parameters optimized in this study^1^.

## 4 Materials and methods

**FIGURE 2:**
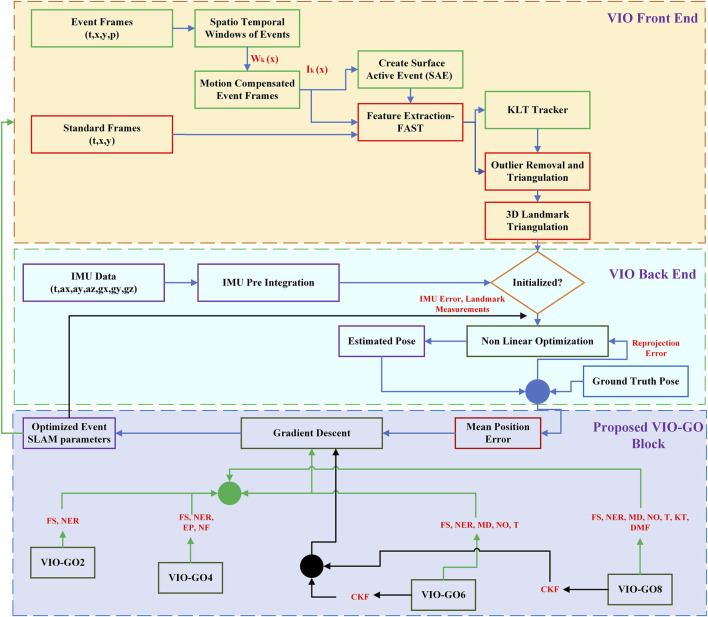
A detailed illustration showcasing the VIO-GO process, highlighting the integration of event frames, standard frames, and IMU using BGD optimization. The figure provides an overview of the iterative optimization process, emphasizing the seamless fusion of event-based visual information and inertial measurements to refine the estimated trajectory and reduce the MPE.

Motion compensation necessitates identifying motion parameters that precisely match a sequence of events. By using a continuous-time warping framework, it is possible to fully leverage the exact temporal information offered by events, setting this approach apart from conventional image-based methods. Obtaining parameters for these continuous-time motion models frequently relies on optimization strategies (Mueggler et al., 2018). This section focuses on optimizing Event SLAM parameters to enhance trajectory pose prediction using the BGD algorithm. A comprehensive diagram of the proposed VIO-GO process flow is depicted in Figure 2. Therefore, BGD is chosen in this study for its proven stability and reliability in achieving efficient parameter tuning.

BGD is chosen due to its fundamental role as an optimization technique widely used in machine learning (Mustapha et al., 2020). Its significance emerges from its ability to systematically uncover optimal parameter values through iterative adjustments guided by gradients of the objective function, computed across the entire dataset. This makes BGD particularly effective for smaller datasets, such as ours, where the dataset is dynamically generated as the robot navigates through the environment. Unlike SGD, which updates parameters based on individual data points and can introduce noise, BGD provides stable convergence, minimizing variance and ensuring more consistent results (Singh and Singh, 2023). Furthermore, successful applications of BGD in parameter optimization, such as in (Mustapha et al., 2020; Rao et al., 2023), demonstrate its robustness and effectiveness in enhancing model performance. Therefore, BGD is chosen in this study for its demonstrated stability and reliability in achieving efficient parameter tuning.

A key challenge with the GD method is that the search may oscillate within the search space, influenced by the gradient’s direction. For instance, although the descent can move toward a global minimum, it may sometimes veer off course due to local minima or saddle points, ultimately slowing convergence. To address this, a common solution is to introduce momentum into the parameter update equation. This approach introduces an additional hyperparameter that controls the extent to which the past gradient (momentum) influences the current update (Chandra et al., 2022). Momentum helps the search maintain a consistent direction, reducing oscillations and enhancing the likelihood of bypassing local minima. In this work, momentum has been added to the BGD algorithm, formulated as shown in Equation 7:

$$G_{i}=\beta *G_{i-1}+g_{i},$$ (7)

where $G_{i}$ defines the adjusted gradient incorporating momentum, β is the hyperparameter that represents the momentum constant, and $g_{i}$ denotes the gradient, showing the direction of decrease for the cost function.

Identifying the optimal event window size is crucial for event-based SLAM systems that use motion compensation to represent event data. This calibration relies on the event frame’s dynamics, influenced more by camera resolution and scene complexity than by the speed of camera motion (Xiao et al., 2022). The number of events N in every spatiotemporal window must be adjusted based on the scene’s texture density, making it a main optimization target in VIO-GO. The primary goal is to achieve sharp motion blur-free edges, ensuring that the event frame accurately reflects the scene’s layout.

The VIO-GO model is implemented alongside the state-of-the-art VIO method, Ultimate SLAM, chosen for its use of motion-compensated images to represent event data, which requires significant parameter adjustments. VIO-GO functions as an auxiliary technique that automatically finds and updates optimal parameters within the Ultimate SLAM framework.

VIO-GO incorporates several approaches: the 2-parameter set (VIO-GO2), the 4-parameter set (VIO-GO4), the 6-parameter set (VIO-GO6), and the 8-parameter set (VIO-GO8). The parameters selected for each approach are determined from the front-end and back-end equations discussed in Section 3. VIO-GO2 and VIO-GO4 concentrate on optimizing the spatiotemporal event window parameters, while VIO-GO6 and VIO-GO8 extend optimization to include feature extraction and back-end parameters. The key parameter sets $\Theta^{\left(0\right)}$ considered for optimizing event VIO are detailed in Table 1.

**TABLE 1:** List of parameter sets $\Theta^{\left(0\right)}$ selected for BGD optimization in event-based VIO.

VIO-GO2		
Parameter	Symbol	Explanation
Frame size	$F_{s}$	Number of events drawn from the event camera
Noise event rate	$N_{ER}$	Events per second regarded as noise

VIO-GO4		
Parameter	Symbol	Explanation
Frame size	$F_{s}$	Number of events drawn from the event camera
Noise event rate	$N_{ER}$	Events per second regarded as noise
Data size augmented event packet	$E_{P}$	Event packet size
Frame norm factor	$N_{F}$	Normalization factor for event frames

VIO-GO6		
Parameter	Symbol	Description
Frame size	$F_{s}$	Number of events drawn from the event camera
Noise event rate	$N_{ER}$	Events per second regarded as noise
VIO median depth	$M_{D}$	Median depth of landmarks
Imp detector num octaves	$N_{O}$	Number of pyramid levels for feature extraction
Imp detector threshold	T	Absolute threshold value of the FAST detector
VIO numkeyframes	$C_{KF}$	Number of keyframes in back-end process

VIO-GO8		
Parameter	Symbol	Description
Frame size	$F_{s}$	Number of events drawn from the event camera
Noise event rate	$N_{ER}$	Events per second regarded as noise
VIO median depth	$M_{D}$	Median depth of landmarks
Imp detector num octaves	$N_{O}$	Number of pyramid levels for feature extraction
Imp detector threshold	T	Absolute threshold value of the FAST detector
VIO numkeyframes	$C_{KF}$	Number of keyframes in the back-end process
VIO kfselect numfts lower thresh	$K_{T}$	Force keyframe selection below this number of features
Detector max features per frame	$D_{MF}$	Maximum number of features to extract per frame

All VIO-GO approaches prioritize the event window size from Equation 1 as the main optimization parameter, due to its critical role, as discussed previously. Furthermore, each method adjusts the noise event rate, which acts as a threshold for scenarios where the sensor is stationary and produces minimal events. When the event rate falls below this threshold, indicating low activity aside from noise events, the sensor is held in a stationary state. These two parameters are the focus of fine-tuning in VIO-GO2.

The optimal values of $\Theta^{\left(0\right)}$ for the VIO-GO2 are calculated from the set of BGD equations, as shown in Equation 8:

$$\begin{array}{ll} & F_{S}=F_{S}-\left(\gamma *G_{FS}\right)\\ & N_{ER}=N_{ER}-\left(\gamma *\frac{G_{N}}{2}\right), \end{array}$$ (8)

where $G_{FS}$ denotes the gradient frame size and $G_{N}$ indicates the gradient noise event rate.

In VIO-GO4, additional parameters are optimized, including the event packet size, which defines the dimensions of augmented event packets sent to the front-end for rendering event frames, and the normalization factor for event frames. However, in VIO-GO6 and VIO-GO8, these parameters were adjusted, as they were found to have minimal impact on the estimated trajectory of the overall VIO system, as discussed in Section 5.

The optimal values of $\Theta^{\left(0\right)}$ for the VIO-GO4 are calculated from the set of BGD equations, as shown in Equation 9:

$$\begin{array}{ll} & F_{S}=F_{S}-\left(\gamma *G_{FS}\right)\\ & N_{ER}=N_{ER}-\left(\gamma *\frac{G_{N}}{2}\right)\\ & E_{P}=E_{P}-\left(\gamma *1.5*G_{EP}\right)\\ & N_{F}=G_{NF}, \end{array}$$ (9)

where $G_{FS}$ is the gradient frame size, $G_{N}$ corresponds to the gradient noise event rate, $G_{EP}$ corresponds to the gradient event packet size, and $G_{NF}$ is the gradient normalization factor.

To enhance feature identification in Equation 4, both VIO-GO6 and VIO-GO8 adjust the FAST detector threshold and the number of pyramid levels used for feature extraction. Additionally, both methods fine-tune the parameter defining the number of keyframes used in the back-end optimization process.

The optimal values of $\Theta^{\left(0\right)}$ for the VIO-GO6 are calculated from the set of BGD equations, as shown in Equation 10:

$$\begin{array}{ll} & F_{S}=F_{S}-\left(\gamma *G_{FS}\right)\\ & N_{ER}=N_{ER}-\left(\gamma *\frac{G_{N}}{2}\right)\\ & M_{D}=M_{D}-\left(\gamma *G_{MD}\right)\\ & N_{O}=G_{NO}\\ & T=T-\left(\gamma *G_{T}\right)\\ & C_{KF}=C_{KF}-\left(\gamma *G_{\mathrm{CKF}}\right), \end{array}$$ (10)

where $G_{FS}$ and $G_{N}$ remain as in the previous approach, $G_{MD}$ corresponds to the gradient of the median depth, $G_{NO}$ is the gradient of the number of octaves, $G_{T}$ is the gradient threshold, and $G_{\mathrm{CKF}}$ corresponds to the gradient of the number of keyframes in the back-end.

VIO-GO8 includes two additional parameters not found in VIO-GO6: the minimum number of features needed to enforce keyframe selection and the maximum number of features to extract from each frame. These parameters have a considerable effect on the feature extraction process, thereby affecting the overall performance of the VIO system.

The optimal values of $\Theta^{\left(0\right)}$ for VIO-GO8 are calculated from the set of BGD equations, as shown in Equation 11:

Algorithm 1 Proposed parameter set $\Theta^{\left(0\right)}$ of optimization method^1^.

**Input:Initial parameters** $\Theta^{\left(0\right)}$ **, Number of iterations** N **, Learning rate** γ

**Output: Final parameters** $\Theta^{\left(N\right)}$

1. **for** n=0 **to** N−1

2.     estimate $\nabla L\left(\Theta^{\left(n\right)}\right)\leftarrow USLAM\left(trajectory\right)$

3.     compute $\Delta \Theta^{\left(n\right)}=-\nabla L\left(\Theta^{\left(n\right)}\right)$

4. $\Theta^{\left(n+1\right)}≔\Theta^{\left(n\right)}+\gamma \Delta \Theta^{\left(n\right)}$

5. **return** $\Theta^{\left(N\right)}$

$$\begin{array}{ll} & F_{S}=F_{S}-\left(\gamma *G_{FS}\right)\\ & N_{ER}=N_{ER}-\left(\gamma *\frac{G_{N}}{2}\right)\\ & M_{D}=M_{D}-\left(\gamma *G_{MD}\right)\\ & N_{O}=G_{NO}\\ & T=T-\left(\gamma *G_{T}\right)\\ & C_{KF}=C_{KF}-\left(\gamma *G_{\mathrm{CKF}}\right)\\ & K_{T}=K_{T}-\left(\gamma *G_{KT}\right)\\ & D_{MF}=D_{MF}-\left(\gamma *G_{\mathrm{DMF}}\right), \end{array}$$ (11)

where $G_{KT}$ represents the gradient of the maximum number of features per frame and $G_{\mathrm{DMF}}$ denotes the gradient keyframes selection threshold, while all other parameters remain the same as in VIO-GO6.

The loss functions for these parameter sets are calculated based on the mean error and target error of the trajectories obtained from the event-based VIO. The parameters are updated using a learning rate γ of 0.02. Once these parameters are optimized, they are fedback into the event-based VIO, and the resulting trajectories in the x , y , and z directions are recorded along with the MPE. The error is calculated over a 5-s interval, as described in (Rebecq et al., 2017b; Vidal et al., 2018). The complete parameter optimization technique is outlined in Algorithm 1.

Although the Ultimate SLAM involves numerous parameters, restricting their number ensures the practical feasibility of the proposed system. This constraint was chosen for two main reasons. First, the selected parameters are crucial elements of the front-end and back-end equations discussed in Section 3. Second, maintaining a fixed learning rate across all VIO-GO approaches makes it challenging to integrate parameters with significantly different values into the GD equations. Moreover, using various learning rates for different parameters would significantly increase the system’s computational cost.

The proposed algorithm extends its applicability beyond Ultimate SLAM, demonstrating adaptability to a broader range of algorithms. Specifically, the VIO-GO2 and VIO-GO4 approaches are applicable to any event-based VIO system that uses motion-compensated images for event data representation. This compatibility is due to the shared use of a spatio-temporal event window in the front-end processing of these systems. Moreover, VIO-GO6 and VIO-GO8 are designed for seamless integration with event-based systems that specifically use the FAST detector for feature extraction and nonlinear back-end optimization.

## 5 Results

**FIGURE 3:**
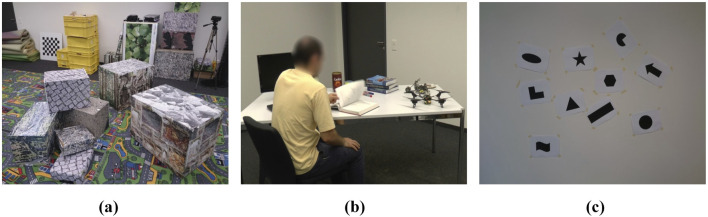
Scenes from the different sequences in the Event Camera Dataset (Mueggler et al., 2017b, Copyright © 2017 by The Author(s). Reprinted by Permission of Sage Publications). **(a)** Boxes_sequence. **(b)** Dynamic_sequence. **(c)** Shapes_sequence.

We assess the efficiency of the VIO-GO framework by comparing it to various event-based VIO methods across challenging sequences from the Event Camera Dataset (Mueggler et al., 2017b). This dataset comprises sequences captured with a Dynamic and Active-pixel Vision Sensor (DAVIS) across various synthetic and real-world environments, serving as a widely accepted benchmark for evaluating SLAM systems for high-speed motion and HDR scenarios. The sequences exhibit complexity through varying speeds, scenes, and DoF. In the shapes, poster, and boxes datasets, each DoF is initially excited individually, followed by mixed and progressively faster excitations, resulting in higher event rates over time. The HDR datasets include significant intrascene contrasts created by a spotlight. The dynamic sequences, gathered in a simulated office environment and observed by a motion-capture method, depict an individual transitioning from sitting at a desk to moving around. Figure 3 displays snapshots from representative sequences within the dataset, highlighting the diversity and complexity of the captured scenarios.

Our evaluation includes a quantitative examination to assess the accuracy of the proposed algorithm. Accuracy is measured using the MPE, expressed as a percentage of the total distance traveled. A 6-DOF transformation in SE(3) is applied over a 5-s segment of the trajectory to align the estimated and ground truth trajectories. This alignment and accuracy calculation is carried out using the EVO tool (Grupp, 2017). All experiments were conducted on a laptop powered by an Apple M1 chip, running Ubuntu 20.04 and ROS Noetic. To evaluate the performance of the presented adaptive optimization system, it was integrated with the Ultimate SLAM framework (Vidal et al., 2018). Ultimate SLAM uses edge images for VIO, requiring significant parameter tuning to adapt to the dynamic nature of events in the scene.

To initiate the BGD optimization process, we set all parameter values to the upper bounds of their respective ranges. This choice provides a conservative starting point, allowing the system to iteratively refine the parameters toward their optimal values. For IMU biases, fixed initial values were used throughout all experiments to ensure consistent benchmarking. These values are derived from the calibration data provided with the Event Camera Dataset Mueggler et al., 2017b. Moreover, they fall within the nominal factory calibration ranges specified in the datasheet of the InvenSense MPU-6150 IMU sensor^2^, which is the integrated IMU sensor in DAVIS. The values used are listed in Table 2.

**TABLE 2:** Initial IMU bias values used in all experiments.

Accelerometer	Value (m/s^2^)	Gyroscope	Value (rad/s)
Bias X	−0.1059	Bias X	0.0494
Bias Y	−0.2015	Bias Y	0.0105
Bias Z	0.2432	Bias Z	0.0012

The evaluation is divided into two parts. First, we analyze various VIO-GO approaches to identify the most effective model, determine the optimal number of parameters for optimization, and test the scalability of the presented approach. Next, in the second part, we compare VIO-GO with the state-of-the-art edge image-based event-driven VIO approaches to highlight its performance advantages.

### 5.1 Evaluating VIO-GO approaches

We evaluate the impact of varying the number of optimized parameters in the VIO-GO approach on the overall performance of the VIO system. This involves comparing VIO-GO2, VIO-GO4, VIO-GO6, and VIO-GO8 across various sequences from the Event Camera Dataset. Table 3 provides a detailed comparison of the results.

**TABLE 3:** The performance of various VIO-GO approaches measured in terms of MPE (%).

Dataset	VIO-GO2 (2 parameters)	VIO-GO4 (4 parameters)	VIO-GO6 (6 parameters)	VIO-GO8 (8 parameters)
boxes_6dof	0.45	0.50	0.44	**0.41**
boxes_translation	0.35	0.27	0.26	**0.25**
dynamic_6dof	0.29	0.35	**0.27**	**0.27**
dynamic_translation	0.33	0.27	0.26	**0.25**
hdr_boxes	0.48	0.46	0.37	**0.35**
hdr_poster	0.29	0.31	0.31	**0.25**
poster_6dof	0.59	0.69	0.54	**0.50**
poster_translation	0.26	0.26	0.25	**0.23**
shapes_6dof	1.05	0.91	**0.77**	**0.77**
shapes_translation	0.64	0.50	**0.33**	0.36
Average	0.47	0.45	0.38	**0.36**

The values displayed in bold show the best results.

The results show that increasing the number of tuned parameters in the proposed model significantly enhances the overall performance of the VIO system. As illustrated in Table 3, optimizing 8 parameters (VIO-GO8) instead of 2 (VIO-GO2) results in a 24% reduction in the average MPE of the estimated trajectory across all sequences. Similarly, VIO-GO4 surpasses VIO-GO2 in most sequences, achieving a 4% reduction in average MPE. Further improvements are observed with VIO-GO6, which reduces the average MPE by 16% compared to VIO-GO4. Finally, VIO-GO8 delivers the most accurate trajectories, achieving an additional 5% reduction in average MPE compared to VIO-GO6 across all tested sequences.

**FIGURE 4:**
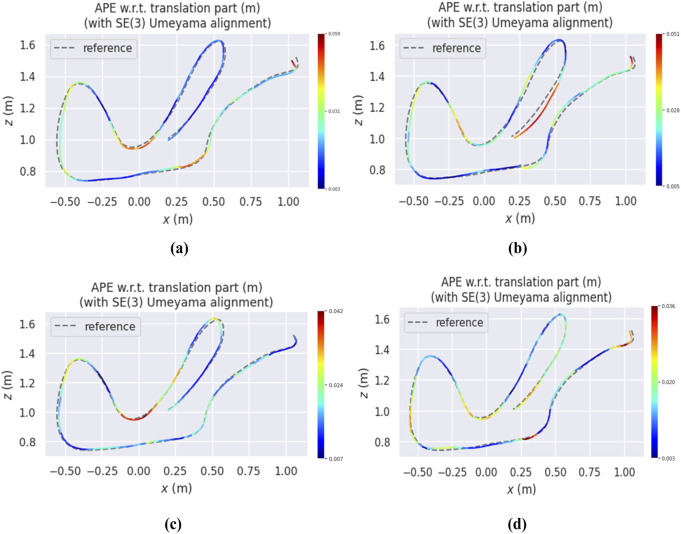
Heatmaps depicting the APE of various VIO-GO trajectories for the *hdr_boxes* sequence, aligned with the ground truth via a 6-DOF transformation over a 5-s period using the EVO tool. **(a)** VIO-GO2. **(b)** VIO-GO4. **(c)** VIO-GO6. **(d)** VIO-GO8.

**FIGURE 5:**
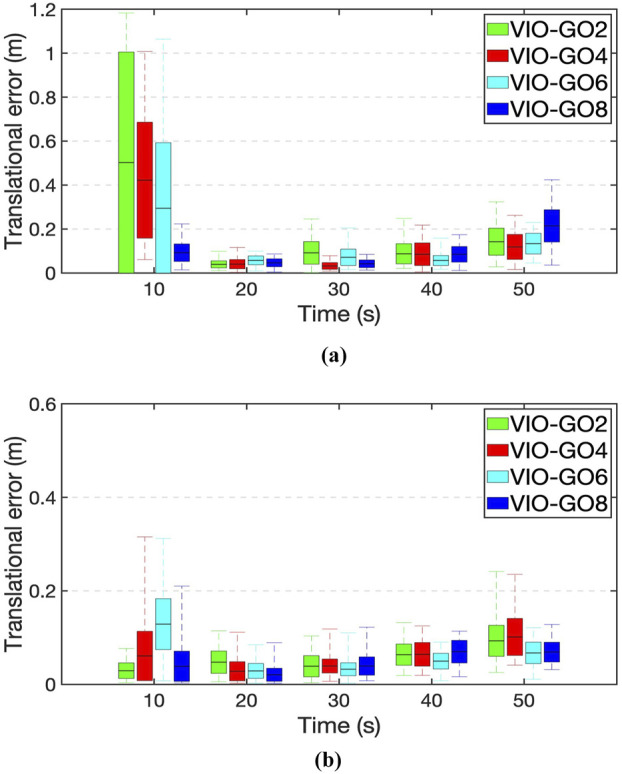
Comparison of relative errors across various VIO-GO variants. **(a)** *Hdr_boxes* sequence. **(b)** *Boxes_translation* sequence.

Figure 4 presents heatmaps of the estimated trajectories obtained from various VIO-GO approaches for the *hdr_boxes* sequence, aligned with the ground truth trajectory. The plots demonstrate that all VIO-GO variants produce precise trajectory estimations, as indicated by the low APE values. Notably, the graphs highlight clear improvement in trajectory accuracy with an increasing number of optimized parameters. This is shown by the significant reduction in the mean APE from 0.031 m for VIO-GO2 to 0.020 m for VIO-GO8. Figure 5 presents relative error metrics to evaluate the performance of different VIO-GO approaches on the *hdr_boxes* and *boxes_translation* sequences. The charts clearly demonstrate the effectiveness of VIO-GO in reducing trajectory drift over time, with notable improvements observed as the number of optimized parameters increases.

To further evaluate computational efficiency, we measured the elapsed time of each VIO-GO configuration (with 2, 4, 6, and 8 parameters) using the same hardware setup. The experiments show that the VIO-GO8 consistently achieves lower elapsed time across most sequences, with an average elapsed time of 16.39 s, compared to 18.98 s for VIO-GO2, 18.51 s for VIO-GO4, and 18.90 s for VIO-GO6. Therefore, an average computational improvement of 13.6% over VIO-GO2 was observed, primarily due to the fine-tuning of key parameters. This computational efficiency gain is achieved during the feature extraction phase, specifically the number of features used to trigger keyframe selection and the maximum number of features extracted per frame. By optimizing these parameters, VIO-GO8 reduces the computational overhead associated with processing redundant or suboptimal features, leading to faster execution. It is important to note that VIO-GO8 requires a higher optimization cost upfront compared to other approaches, due to the increased number of parameters being tuned. Nevertheless, this does not significantly impact the time required to find the optimal parameters, as the BGD algorithm efficiently explores the parameter space in parallel and requires minimal computational resources.

Although VIO-GO8 delivers the best results in terms of both accuracy and computational efficiency, it is worth noting that increasing the number of optimized parameters beyond eight may further enhance performance. However, such an expansion would also introduce greater complexity into the optimization process. As noted previously, the choice to limit the parameter set to eight was driven by practical considerations, including the constraints of maintaining a fixed learning rate and controlling computational overhead associated with parameter tuning. Nonetheless, extending the optimization to a broader set of parameters remains a promising direction for future research.

### 5.2 Comparing with event-based VIO methods

In our evaluation, we benchmark the proposed system against the raw results of Ultimate SLAM, as reported by its authors who used per-sequence parameter tuning and accurate IMU bias initialization. Building upon the Ultimate SLAM framework, our model is evaluated against this baseline to demonstrate its capability to automatically identify optimal parameters for each sequence in the Event Camera Dataset. Additionally, we compare it with Ultimate SLAM results obtained using a fixed parameter set adjusted across all sequences simultaneously and initialized with zero IMU bias, as presented in (Mahlknecht et al., 2022).

The aim of this comparison is to demonstrate the importance of parameter optimization in event-based VIO methods and to highlight the performance of the proposed model against a fixed parameter set across various scenarios. Moreover, we compare VIO-GO with (Rebecq et al., 2017b), an event-based algorithm coupled with an IMU, considered the foundational pipeline for Ultimate SLAM. The evaluation also includes EKLT-VIO (Mahlknecht et al., 2022), a system that integrates the EKLT feature tracker with a filter-based back-end, and EVIO (Zhu et al., 2017), an event-based tracking algorithm combined with an IMU. Similar to the proposed approach, both EKLT-VIO and EVIO are developed to perform efficiently under diverse conditions, including HDR environments and different lighting scenarios. The developers of the selected EVIO methods evaluated them using MPE as the error metric and the Event Camera Dataset as the simulation environment, following the same evaluation methodology employed in this work.

Table 4 provides a comprehensive comparison of the MPE five benchmark algorithms and VIO-GO using the 8-parameter configuration (VIO-GO8), across various sequences from the Event Camera Dataset. As shown in Table 4, the presented integrated system achieves state-of-the-art performance. Compared to Ultimate SLAM with a fixed parameter set (Vidal et al., 2018; Mahlknecht et al., 2022), which has an average MPE of 0.89%, VIO-GO8 demonstrates superior performance across all sequences with an average MPE of 0.36%. In contrast to the raw results of Ultimate SLAM (Vidal et al., 2018), VIO-GO8 successfully identifies optimal parameters, resulting in a lower MPE in the *boxes_translation*, *hdr_boxes*, and *hdr_poster* sequences, with MPE values of 0.25%, 0.35%, and 0.25%, respectively. Although the raw results of Ultimate SLAM exhibit better performance compared to VIO-GO, it is important to note that Ultimate SLAM heavily relies on manual parameter tuning for each sequence, which is considered impractical. Conversely, VIO-GO automatically fine-tunes the selected parameters across different environments. Furthermore, as explained in Section 4, we opted for only eight key parameters that we identified as directly influencing the system performance. In contrast, Ultimate SLAM has a much larger set of parameters that can be adjusted for improved results, but this comes at the cost of requiring substantial computational time. For these reasons, we have grayed-out the Ultimate SLAM results in Table 4. This decision to downplay Ultimate SLAM was made to highlight the practical advantages of our simpler parameter set over the more computationally intensive Ultimate SLAM, thereby focusing on the efficiency and practicality of VIO-GO in real-world scenarios. Notably, VIO-GO8 surpasses all other approaches in 7 out of 10 sequences. With an average MPE of 0.36%, VIO-GO8 exhibits a 16% reduction in MPE compared to the 0.43% reported in (Rebecq et al., 2017b), a 33% lower MPE than EKLT-VIO (Mahlknecht et al., 2022) with 0.54% MPE, and an 86% lower MPE compared to EVIO (Zhu et al., 2017), which reports an MPE of 2.57%.

**TABLE 4:** Performance of the proposed VIO-GO against other event-based VIO systems in terms of MPE in %. The USLAM* results are obtained by individually tuning parameters for each sequence, whereas Fixed USLAM uses a single set of parameters that is tuned across all sequences simultaneously.

Dataset	USLAM^a^ Vidal et al. (2018)	Fixed USLAM	Rebecq et al. (2017b)	EKLT-VIO Mahlknecht et al. (2022)	EVIO Zhu et al. (2017)	VIO-GO8 (8 parameters)
boxes_6dof	0.30	0.68	**0.36**	0.84	3.61	0.41
boxes_translation	0.27	1.12	0.31	0.48	2.69	**0.25**
dynamic_6dof	0.19	0.76	0.56	0.79	4.07	**0.27**
dynamic_translation	0.18	0.63	0.39	0.40	1.90	**0.25**
hdr_boxes	0.37	1.01	0.59	0.46	1.23	**0.35**
hdr_poster	0.31	1.48	0.33	0.65	2.63	**0.25**
poster_6dof	0.28	0.59	0.40	**0.35**	3.56	0.50
poster_translation	0.12	0.24	0.46	0.35	0.94	**0.23**
shapes_6dof	0.10	1.07	**0.42**	0.60	2.69	0.77
shapes_translation	0.26	1.36	0.50	0.51	2.42	**0.36**
Average	0.24	0.89	0.43	0.54	2.57	**0.36**

^a^ Requires substantial parameter adjustments based on the dynamic events in the scene.

The values displayed in bold show the best results.

**FIGURE 6:**
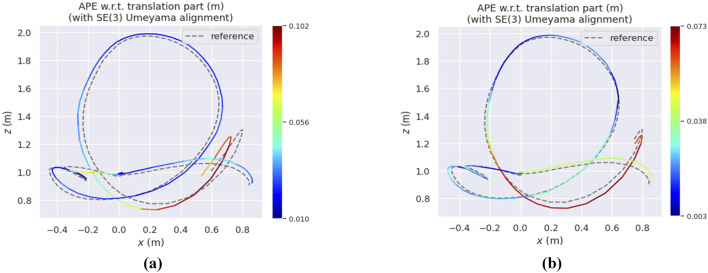
Heatmaps presents the APE for the VIO-GO trajectory and the Ultimate SLAM raw trajectory for the Boxes_6dof sequence, both aligned with the ground truth trajectory using a 6-DOF transformation in SE3 over a 5-s duration, as generated by the EVO tool. **(a)** VIO-GO evaluated on *Boxes_6dof*. **(b)** USLAM* evaluated on *Boxes_6dof*.

**FIGURE 7:**
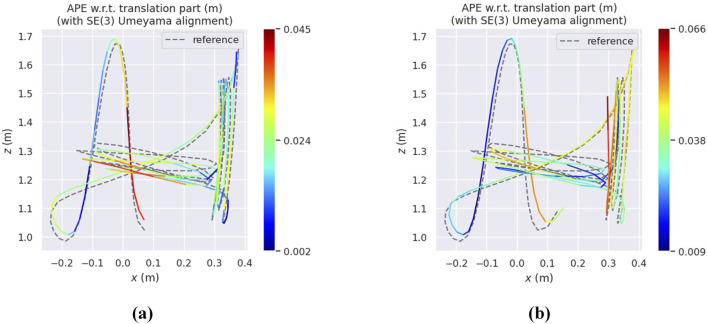
Heatmaps presents the APE for the VIO-GO trajectory and the Ultimate SLAM raw trajectory for the Boxes_Translation sequence, both aligned with the ground truth trajectory using a 6-DOF transformation in SE3 over a 5-s duration, as generated by the EVO tool. **(a)** VIO-GO evaluated on *Boxes_translation*. **(b)** USLAM* evaluated on *Boxes_translation*.

**FIGURE 8:**
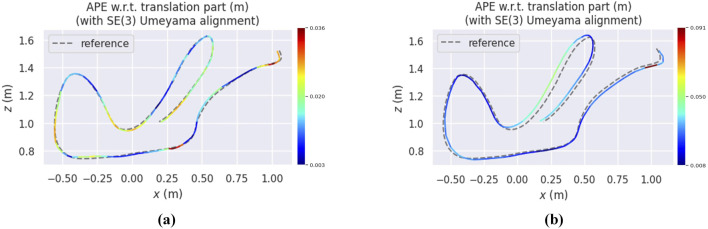
Heatmaps presents the APE for the VIO-GO trajectory and the Ultimate SLAM raw trajectory for the Hdr_Boxes sequence, both aligned with the ground truth trajectory using a 6-DOF transformation in SE3 over a 5-s duration, as generated by the EVO tool. **(a)** VIO-GO evaluated on *Hdr_boxes*. **(b)** USLAM* evaluated on *Hdr_boxes*.

Figures 6–8 illustrate heatmaps of the estimated trajectories from the proposed approach alongside the raw trajectory from Ultimate SLAM, both aligned with the ground truth trajectory for three different sequences from the Event Camera Dataset. In Figure 6, which corresponds to the boxes_6dof sequence, VIO-GO8 demonstrates high trajectory accuracy, closely aligning with the ground truth and achieving a low APE. However, its APE is slightly higher than that of Ultimate SLAM. This difference is primarily attributed to Ultimate SLAM relying on extensive manual tuning across a wide range of parameters. In contrast, VIO-GO automatically optimizes a fixed subset of eight key parameters. While broader manual tuning can improve accuracy, it increases system complexity and limits scalability. VIO-GO prioritizes efficiency and generalizability by eliminating the need for manual intervention. Figures 7, 8 present results for the *hdr_boxes* and *boxes_translation* sequences, respectively. In both cases, VIO-GO8 outperforms Ultimate SLAM by producing trajectories that more closely align with the ground truth and achieving lower APE values. These improvements highlight VIO-GO’s ability to adapt parameter configurations to challenging conditions without requiring manual tuning. The results further demonstrate the robustness and flexibility of the proposed framework across diverse scenarios.

**FIGURE 9:**
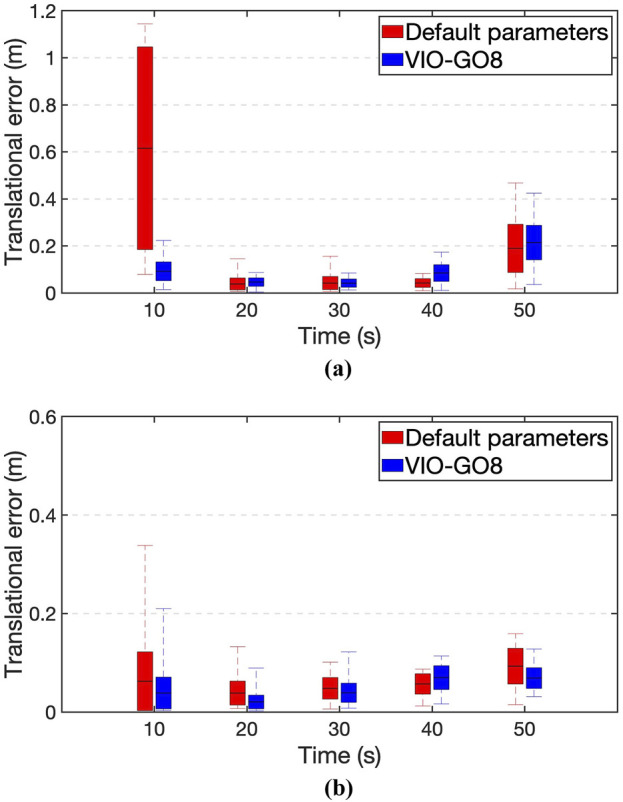
The relative error comparison between Ultimate SLAM with its default parameters and VIO-GO8 with its optimized parameters. **(a)** *Hdr_boxes* sequence. **(b)** *Boxes_translation* sequence.

In Figure 9, we employ relative error metrics to compare VIO-GO8 to Ultimate SLAM with its default parameter configuration applied to the *hdr_boxes* and *boxes_translation* sequences. The results show that VIO-GO8 notably reduces drift in the estimated trajectory over time. Table 5 presents a time analysis comparison between Ultimate SLAM, using its default parameters, and VIO-GO8 with its optimal parameter set. As shown, VIO-GO8 requires significantly less time to process all datasets compared to Ultimate SLAM. This performance improvement is attributed to VIO-GO8’s ability to dynamically select the best parameter set for each sequence, thereby reducing processing overhead in both the front-end and back-end stages. Furthermore, as previously discussed, VIO-GO8 outperforms the fixed parameter set approach by achieving an average MPE that is 58% lower than Ultimate SLAM’s default parameters across all sequences.

**TABLE 5:** Elapsed time comparison between Ultimate SLAM with its default parameters and VIO-GO8 with its optimized parameters.

Dataset	USLAM (default parameters)		VIO-GO8	
	Time cost (s)	MPE (%)	Time cost (s)	MPE (%)
boxes_6dof	20.01	0.49	**18.56**	**0.41**
boxes_translation	22.11	0.38	**20.30**	**0.25**
dynamic_6dof	17.83	0.66	**14.50**	**0.27**
dynamic_translation	17.65	1.07	**10.94**	**0.25**
hdr_boxes	20.58	1.12	**18.39**	**0.36**
hdr_poster	21.47	0.51	**18.83**	**0.25**
poster_6dof	22.14	0.96	**21.90**	**0.50**
poster_translation	18.71	0.35	**16.18**	**0.23**
shapes_6dof	16.45	1.46	**11.30**	**0.77**
shapes_translation	15.35	0.70	**12.99**	**0.36**
Average	19.23	0.77	**16.39**	**0.36**

The values displayed in bold show the best results.

## 6 Discussion

In this section, we highlight the effectiveness of VIO-GO in addressing key challenges such as parameter optimization and computational efficiency. Additionally, we reflect on the broader impact of our findings, explore potential research avenues, and identify areas for improvement to guide future advancements in this field.

### 6.1 Contributions

The primary contribution of VIO-GO is its ability to automatically optimize parameters for event-based VIO systems, significantly improving both accuracy and computational efficiency. Specifically, the VIO-GO8 approach, which optimizes eight key parameters, achieves an average MPE of 0.36%, outperforming fixed-parameter approaches such as Ultimate SLAM and other state-of-the-art methods, including EKLT-VIO and EVIO. These results underline the effectiveness of adaptive parameter tuning in enhancing VIO performance across diverse and dynamic environments.

A critical observation is the scalability of VIO-GO, where system performance improves with the inclusion of additional optimized parameters. For instance, a comparison between VIO-GO2 and VIO-GO8 demonstrates the benefits of comprehensive parameter optimization. Moreover, VIO-GO eliminates the need for manual parameter tuning required by previous methods, significantly reducing deployment time and effort. This makes it particularly well-suited for applications in Industry 4.0, where environments are highly variable and demand rapid adaptation. The core design of VIO-GO emphasizes generalizability. By dynamically optimizing a fixed set of key parameters based on scene characteristics, it adapts automatically to diverse conditions without relying on predefined configurations. In contrast to traditional motion-compensation approaches that require extensive manual adjustment for each new environment, VIO-GO offers a more scalable and practical solution.

In comparison with existing approaches, VIO-GO introduces a paradigm shift by automating the parameter optimization process. Our results show that VIO-GO significantly reduces trajectory drift over time and achieves a lower APE compared to fixed-parameter approaches. This is crucial for real-time applications, making VIO-GO an ideal candidate for resource-constrained scenarios in industrial robotics and autonomous navigation.

### 6.2 Limitations

While VIO-GO demonstrates promising results, several limitations remain. One key challenge is its dependency on a predefined set of key parameters, which may constrain its adaptability to highly diverse or previously unseen environments. Future iterations could expand the parameter set or incorporate environment-specific variables, allowing the system to adapt more effectively to complex scenarios. Another limitation is the sensitivity of the system to initial conditions, such as IMU bias and feature selection, which may affect stability during extended operations. Future efforts could address these challenges through advanced initialization methods and noise mitigation strategies.

Additionally, while the Event Camera Dataset provides a valuable and well-calibrated benchmark for evaluating event-based VIO systems, it represents a relatively controlled environment. In real-world scenarios, factors like unstructured environments, sensor noise, and erratic motion patterns can significantly affect event data quality. VIO-GO is designed to address such variability through its core capability of dynamically optimizing key system parameters based on the characteristics of each scene. This allows the system to adapt in real time without requiring manual reconfiguration. Nevertheless, transferring the system from a controlled dataset to real-world deployment may affect the effectiveness of the selected parameter sets. Real-world conditions could present edge cases or variations not fully represented in the dataset, potentially impacting the convergence behavior or responsiveness of the optimization process. For instance, parameters such as the frame size and noise event rate might need adjustments to account for fluctuating event densities caused by background activity. Furthermore, parameters related to feature extraction may need to be tuned to handle less structured or more repetitive textures commonly found in natural scenes. These factors underscore that testing VIO-GO in real-world environments would provide a deeper understanding of its robustness in diverse and unpredictable conditions. Lastly, the use of BGD for parameter optimization, while effective, could be complemented by exploring alternative techniques, such as SGD, Bayesian optimization, or Gauss-Newton methods, to improve convergence speed and efficiency.

### 6.3 Future directions

Building on the current success of VIO-GO, several promising research avenues could extend its capabilities:

1. Expansion of the Parameter Optimization Scope: Extending the optimization to a larger set of parameters remains a promising avenue for future work. While this study limited the number of optimized parameters to maintain practical feasibility, expanding this scope could potentially unlock additional performance improvements.
2. Integration with Other Event-Based SLAM Approaches: Future work could explore extending VIO-GO to integrate with other event-based SLAM systems. This would help develop more robust solutions adaptable to a wider range of applications.
3. Exploration of Advanced Event Processing Techniques: Future studies could look into advanced event-based processing techniques, including deep learning-based methods for event-to-image conversion or more sophisticated feature tracking approaches. These could further boost the performance of event-based VIO systems.
4. Real-Time Adaptation and On-the-Fly Tuning: Implementing real-time adaptation and on-the-fly parameter tuning would make VIO-GO more suitable for autonomous systems operating in unpredictable environments, minimizing the need for pre-set parameters.

By addressing these limitations and expanding the scope of the study, future research could significantly advance the field of Event SLAM, contributing to the development of more robust, efficient, and adaptable systems for autonomous navigation in dynamic environments.

## 7 Conclusion

This work presents VIO-GO, a novel framework for automated parameter optimization in event-based VIO systems, tailored for use in dynamic environments central to Industry 4.0 applications. Designed to address the challenges of dynamic and variable environments, VIO-GO achieves a balance of accuracy and computational efficiency by using motion-compensated images and a BGD algorithm, enhancing the performance and robustness of Event SLAM systems.

Our evaluation on the Event Camera Dataset shows that VIO-GO outperforms fixed-parameter approaches, achieving a 60% reduction in MPE. The system successfully identifies optimal parameters for Ultimate SLAM across multiple sequences, confirming its adaptability to scenarios characterized by fluctuating event rates. This capability is particularly critical for industrial applications, where environmental variability demands highly responsive and efficient navigation solutions.

These results highlight the importance of automated parameter optimization in event-based SLAM systems. Future research should focus on testing VIO-GO in more diverse and complex real-world settings, incorporating advanced event-based processing techniques and exploring alternative optimization methods to further enhance performance. Additionally, VIO-GO’s adaptability can be further evaluated across a wider range of datasets and integrated with other event-based SLAM approaches beyond Ultimate SLAM, expanding its applicability and generalizability to real-world scenarios. By addressing these directions, VIO-GO could establish a new standard for robust, scalable, and adaptive SLAM solutions, particularly in the demanding contexts of Industry 4.0 and beyond.

## Data Availability

The original contributions presented in the study are included in the article/supplementary material, further inquiries can be directed to the corresponding author.

## Footnotes

1 Refer to Table 1 for key parameters used in the state estimation framework and optimized through the proposed method outlined in Section 4.

2 IMU datasheet: https://www.cdiweb.com/datasheets/invensense/ps-mpu-6100a
